# Uterine Cervical Change at Term Examined Using Ultrasound Elastography: A Longitudinal Study

**DOI:** 10.3390/jcm10010075

**Published:** 2020-12-28

**Authors:** Hyun Soo Park, Hayan Kwon, Ja-Young Kwon, Yun Ji Jung, Hyun-Joo Seol, Won Joon Seong, Hyun Mi Kim, Han-Sung Hwang, Ji-Hee Sung, Soo-young Oh

**Affiliations:** 1 Department of Obstetrics and Gynecology, Dongguk University Ilsan Hospital, Dongguk University, Goyang 10326, Korea; 2 Division of Maternal-Fetal Medicine, Department of Obstetrics and Gynecology, Institute of Women’s Medical Life Science, Yonsei University College of Medicine, Yonsei University Health System, Seoul 03722, Korea; whitekwonmd@gmail.com (H.K.); jaykwon@yuhs.ac (J.-Y.K.); ccstty@yuhs.ac (Y.J.J.); 3 Department of Obstetrics and Gynecology, Kyung Hee University Hospital at Gangdong, Seoul 05278, Korea; seolhj@khu.ac.kr; 4 Department of Obstetrics and Gynecology, Kyungpook National University Hospital, Daegu 41944, Korea; wjseong@knu.ac.kr (W.J.S.); hyunmik@gmail.com (H.M.K.); 5 Department of Obstetrics and Gynecology, Konkuk University Medical Center, Konkuk University School of Medicine, Seoul 05030, Korea; hwanghs@kuh.ac.kr; 6 Samsung Medical Center, Department of Obstetrics and Gynecology, Sungkyunkwan University School of Medicine, Seoul 06351, Korea; obgysung@gmail.com (J.-H.S.); ohsymd@skku.edu (S.-y.O.)

**Keywords:** ultrasonography, elastography, uterine cervix, term pregnancy, parturition

## Abstract

The aim of the study was to investigate if there are changes in elastographic parameters in the cervix at term around the time of delivery and if there are differences in the parameters between women with spontaneous labor and those without labor (labor induction). Nulliparous women at 36 weeks of gestation eligible for vaginal delivery were enrolled. Cervical elastography was performed and cervical length were measured using the E-Cervix^TM^ system (WS80A Ultrasound System, Samsung Medison, Seoul, Korea) at each weekly antenatal visit until admission for spontaneous labor or labor induction. E-Cervix parameters of interest included elasticity contrast index (ECI), internal os strain mean level (IOS), external os strain mean level (EOS), IOS/EOS strain mean ratio, strain mean level, and hardness ratio. Regression analysis was performed using days from elastographic measurement at each visit to admission for delivery and the presence or absence of labor against cervical length, and each E-Cervix parameter fitted to a linear model for longitudinal data measured repeatedly. A total of 96 women were included in the analysis, (spontaneous labor, *n* = 39; labor induction, *n* = 57). Baseline characteristics were not different between the two groups except for cesarean delivery rate. Cervical length decreased with advancing gestation and was different between the two groups. Most elastographic parameters including ECI, IOS, EOS, strain mean, and hardness ratio were significantly different between the two groups. In addition, ECI, IOS, and strain mean values significantly increased with advancing gestation. Our longitudinal study using ultrasound elastography indicated that E-cervix parameters tended to change linearly at term near the time of admission for delivery and that there were differences in E-Cervix parameters according to the presence or absence of labor.

## 1. Introduction

Human parturition begins with structural and biochemical changes in the uterus, including the uterine body and cervix. The pregnant cervix becomes soft from early pregnancy and ripens shortly before labor through the biochemical changes [1]. Such biochemical change during cervical ripening include decreased collagen concentration, increased hydrophilic glycosaminoglycans, hyaluronic acid, and water [2]. Extensive remodeling before labor subsequently leads to uterine cervical effacement and dilatation from forceful uterine contractions.

Since the report of association between short cervical length and increased risk of preterm delivery, cervical length measurement has been used to predict spontaneous preterm delivery [3]. However, as the prevalence of high-risk population is low, and cervical length measurement has low sensitivity to predict preterm birth, the utility of cervical length measurements has been limited. In addition, some researchers reported considerable inter-observer variability [4,5] and inadequate measurement of cervical length, especially when using transabdominal ultrasound, which limits the utility of cervical length measurement as a screening tool [6]. Therefore, researchers are investigating various methods to detect biomechanical or biochemical changes of the cervix to improve the ability to predict spontaneous preterm delivery using ultrasonography, which includes quantitative cervical texture analysis (mean gray level histogram) and elastography.

Quantitative cervical texture analysis uses a histogram with different indices such as mean gray level [7], mean gray-scale values [8] or cervical texture-based score [9] from gray-scale ultrasound image of the uterine cervix. Some of them report promising results in predicting spontaneous preterm birth [8,9]. Recently, ultrasound elastography has been introduced to assess the elasticity or compressibility of tissues for the diagnosis of breast cancer and liver fibrosis [10]. In the obstetrical field, elastography has been studied in the uterine cervix, and the diagnostic performance has been validated in predicting successful induction of labor and preterm delivery in low and high-risk pregnancies [11]. All these efforts to predict labor focus on the changes in the elastic or biomechanical properties of the cervix, and that is based on the premise that elastic changes reflect the biochemical changes inside the cervix. The traditional way to evaluate cervical change is to measure the cervical length, which has been used for the prediction of preterm birth and successful labor induction [12,13]. However, cervical length measurements only detect changes in the physical dimension of the cervix. Detecting biomechanical and biochemical changes before labor could help us understand and predict the common labor processes in preterm or term pregnancy. There are many studies looking into preterm delivery prediction using elastography, and there is a recent report regarding elastographic changes in each trimester [14]. On the other hand, there is a paucity of information regarding immediate changes in the cervix near term parturition.

E-Cervix^TM^ elastography is one of the strain elastographic ultrasound applications that gets compression signals using internal or in vivo compression forces such as adjacent arterial pulsation and breathing. As it uses internal compression, E-Cervix elastography is less operator-dependent than other ultrasound elastography machines that employ external compression. We have reported its reproducibility and utility in predicting preterm delivery in pregnancies with a moderately short cervix [15,16,17]. We found that there were changes in E-Cervix parameters antedating preterm delivery. However, our understanding of the temporal cervical change is poor, especially in term cervix around the time of labor. Therefore, we planned a longitudinal study to investigate if we could detect cervical changes in the strain as well as the length using E-Cervix elastography. We also wanted to look at the differences in the elastographic parameters in the cervix between spontaneous labor and no labor (labor induction) groups. The aim of the study was to investigate if there were changes in elastographic parameters in the cervix at term around the time of delivery and if there were differences in the parameters between women with spontaneous labor and those without labor (labor induction).

## 2. Materials and Methods

### 2.1. Subjects

Between July 2019 and April 2020, nulliparous women eligible for vaginal delivery were invited to participate in this study. They were enrolled at 36 weeks of gestation from three institutions (Dongguk University Ilsan Hospital, Konkuk University Medical Center, and Kyung Hee University Hospital at Gangdong). Four investigators (H.-J.S., H.K., H.-S.H., H.S.P.) who had experience in the use of E-Cervix elastography for more than three years performed cervical elastography. Ultrasound elastography was carried out by one operator in each patient. Cervical elastography and cervical length were measured at each weekly antenatal visit until admission for spontaneous labor or labor induction. Labor was induced according to the managing clinician’s discretion. Multiple pregnancies or pregnancies with cerclage were excluded from this study. Pregnancy outcomes, demographic data, and obstetric data were collected. The study protocol was reviewed and approved by the Institutional Review Board of each participating hospital. Written informed consent was collected from all women.

### 2.2. Cervical Length and Elastographic Measurements

We performed cervical elastography with a vaginal ultrasound (WS80A Ultrasound System, Samsung Medison, Seoul, Korea) using a 6-MHz transvaginal probe. After measuring cervical length, elastography was performed three times in the same plane with the same transvaginal probe using an E-Cervix^TM^ system. The median values of the three measurements were used for the analysis. The cervical length measurement and E-Cervix elastography were performed according to previously described protocol [17]. E-Cervix uses in vivo compression by internally generating fine vibration through organ motion, such as adjacent arterial pulsation and breathing without manual compression. The E-Cervix parameters included in the analysis were elasticity contrast index (ECI), internal (IOS) and external os (EOS) of cervix strain mean level, IOS/EOS strain mean ratio, strain mean level, and hardness ratio (Table 1).

### 2.3. Plots and Regression Analysis

Spaghetti plots were depicted to visualize the serial changes of the cervical length and E-Cervix parameters. Each measurement from an individual was connected and plotted against days from measurements to admission. Fitted lines from linear regression were drawn to represent serial changes in the parameters. We also made the lasagna plot, which is a heatmap for longitudinal data where each subject’s trajectory over time is a horizontal layer, with the simultaneous plotting of trajectories resulting in the stacking of layers, as in lasagna [18].

**Figure 1:**
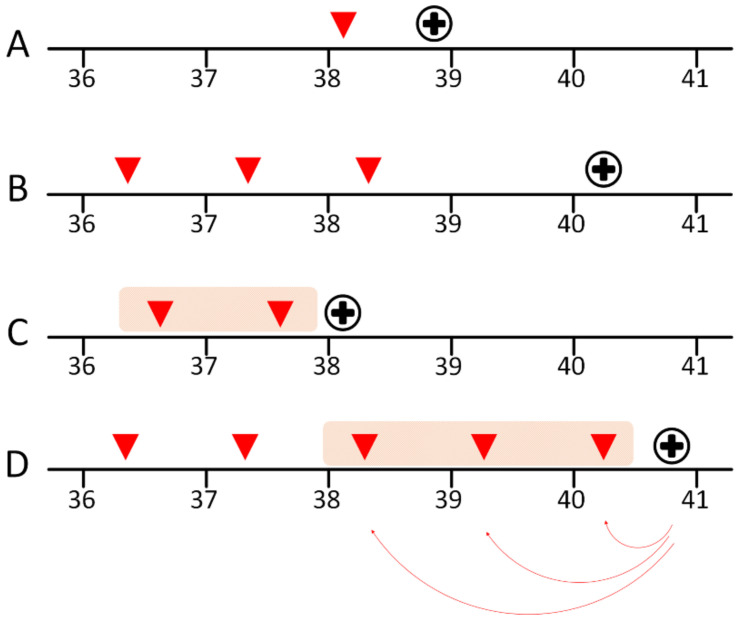
Scheme of measurements. From 36 weeks of gestation, E-Cervix was measured at each weekly visit until admission for spontaneous labor or labor induction. Days from admission to measurements were counted and used for analysis. Subjects whose last measurement before delivery was within seven days and measurements within three weeks were included in the analysis (data in the shaded area in cases of C and D). As elastography was measured only once in case A, and the last measurement was more than seven days from the admission in case B, data were not included in the analysis. Numbers, weeks of gestation; Red arrowhead, time of each measurement; Black cross in a circle, admission for delivery; Red curved arrow, calculation of days from measurement to admission (e.g., −4. −11, and −18); Shades, data used in the analysis.

As we were interested in the elastographic changes of the cervix near the time of the delivery, subjects whose last measurement before delivery was within seven days and measurements within three weeks from the admission were included in the analysis. We hypothesized that E-Cervix parameters would change over time, and there would be differences in the elastographic parameters between the spontaneous labor and labor induction groups. To test the hypothesis, we performed regression analysis using days from elastographic measurement at each visit to the admission for the delivery (Days, Figure 1) and the presence or absence of the labor (Labor) against cervical length and each E-Cervix parameter (y) using the following equation: y = ß0+ ß1* Days + ß2* Labor group. To evaluate the changes of the parameters with time only, we also performed regression analysis using the equation: y= α0 + α1*Days. We used a linear model for longitudinal data by employing mixed procedure in SAS statistical analysis packages, which enables us to handle covariances [19].

### 2.4. Other Statistical Analysis

Maternal baseline characteristics and obstetric outcomes were collected and compared between the spontaneous labor and labor induction groups. Continuous variables were compared using Mann–Whitney U tests. Categorical variables were compared using Fisher’s exact tests. A *p*-value of less than 0.05 was considered significant. STATA 14.0 (StataCorp LLC, College Station, TX, USA) and SAS 9.3 (SAS Institute Inc., Cary, NC, USA) were used for statistical analyses.

## 3. Results

**Figure 2:**
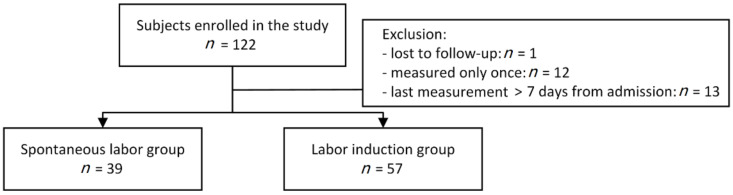
Flow diagram showing the number of subjects enrolled in this study.

During the study period, a total of 122 women were enrolled. Of those, elastography was performed only once in 12 patients. The last measurement was more than seven days before the day of admission in 13 patients. One was not followed up. Finally, 96 women were included in the analysis (spontaneous labor, *n* = 39; labor induction, *n* = 57) (Figure 2). The elastography was performed 2, 3, and 4 times in 27 (28.1%), 64 (66.7%), and 5 (5.2%) patients, respectively. Indications for labor induction were as follows: post-term pregnancy (31.6%, 18/57), pre-labor rupture of membranes (24.6%, 14/57), maternal request (14.0%, 8/57), large for gestational age (7.0%, 4/57), hypertensive disorders (5.3%, 3/57), oligohydramnios (5.3%, 3/57), fetal growth restriction (5.3%, 3/57), and others (7.2%, 4/57).

The baseline characteristics of the participants and pregnancy outcomes are shown in Table 2. The mean maternal age in spontaneous labor and labor induction group was 31.9 (range, 24–39) and 33.4 (range, 24–41), respectively. The cesarean delivery rate was higher in the labor induction group than in the spontaneous labor group. Hypertensive diseases were found in 5.26% of the labor induction group only but were not statistically different. The frequency of pregnancies with in vitro fertilization-embryo transfer (IVF-ET) was 20.8% (20/96). Gestational diabetes consisted of 13.5% (13/96) of the study population. Other variables did not show a statistically significant difference between the two groups.

Table 3 shows the regression results against cervical length and the E-Cervix parameters using days from the measurement to admission with or without spontaneous labor (labor induction and spontaneous labor). When only days from the measurements to admission were put in the regression analysis, ECI, IOS strain mean level, and strain mean significantly increased with advancing gestation along with cervical length. In the model including both days from the measurements to admission and labor groups, ECI, IOS strain mean level, and strain mean again significantly increased with advancing gestation, and they also were significantly different between the two groups. ECI, IOS strain, and strain mean increased by 0.122 and 0.011 and 0.008 per week three weeks from the admission, respectively. EOS and hardness ratio were different between the two groups. Although the hardness ratio decreased with time, such a decrease did not reach statistical significance. With this table, we can explain the change of the cervix according to time and labor group. If we take the example with cervical length, we can say that “Cervical length of the singleton pregnancy at term will decrease by 0.02468 cm everyday within 3 weeks before the admission for delivery in both spontaneous labor and labor induction group. In addition, the cervical length of the spontaneous labor group is 0.481 cm shorter than that of labor induction group throughout the period.” Other parameters can be interpreted in the same manner.

**Figure 3:**
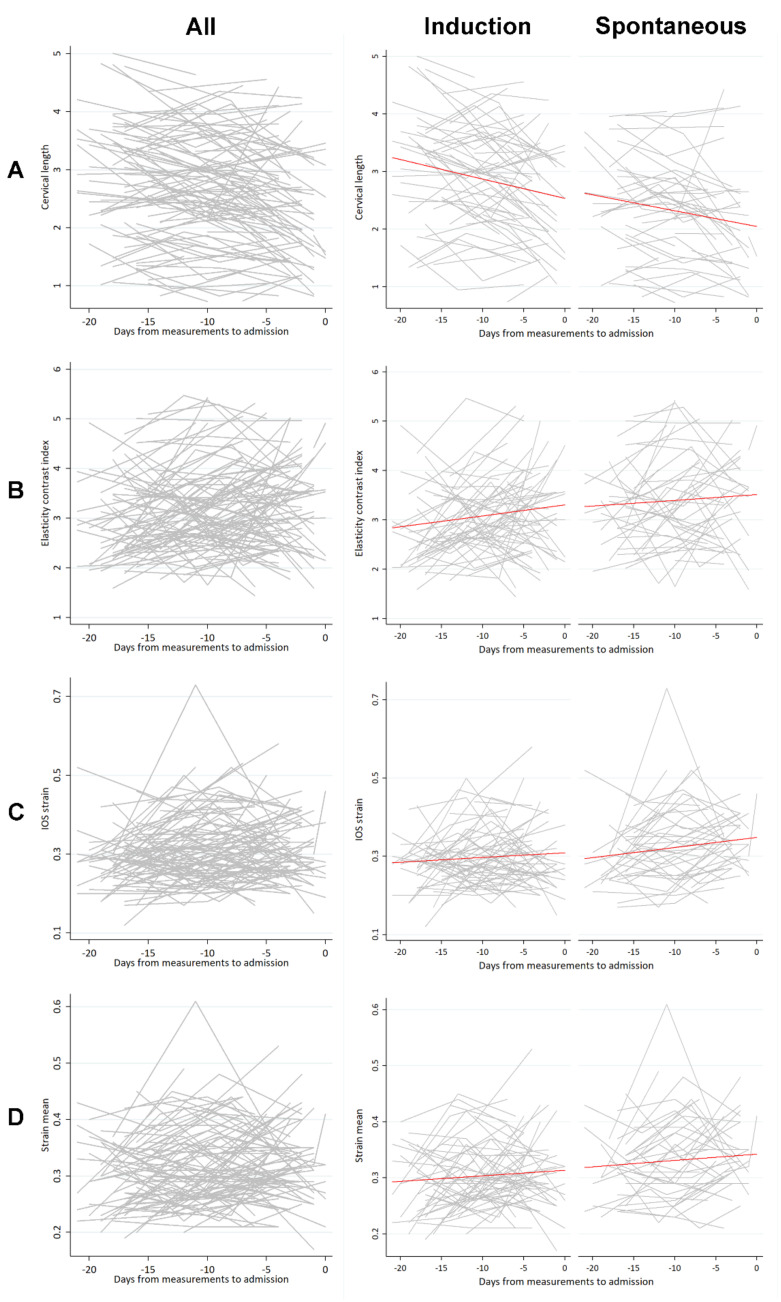
Spaghetti plots of the cervical length and the selected E-Cervix parameters against days from measurement to admission. The number on the *x*-axis represents days from admission (indicated as 0), and the number on the *y*-axis represents the value of cervical length and each E-Cervix parameter. Red lines in the middle and right column indicate line fitted by linear regression. (**A**): Cervical length; (**B**): Elasticity contrast index; (**C**): Strain mean of the internal os of the cervix; (**D**): Strain mean level; **Left** column, plots including all subjects; **Middle** column, plots of the labor induction group; **Right** column, plots of the spontaneous labor group.

Figure 3 shows the combination of the spaghetti plots that connect cervical length and E-Cervix parameter measurements at each visit for each individual. In the middle and right columns, the measured values were fitted in a regression equation to generate red lines illustrating the overall levels of the values in the labor induction and spontaneous labor groups and the change of the parameters with time. For example, the parameter of ECI tended to increase with time, and the levels of the parameter were different between spontaneous labor and labor induction groups, reflecting the regression results.

**Figure 4:**
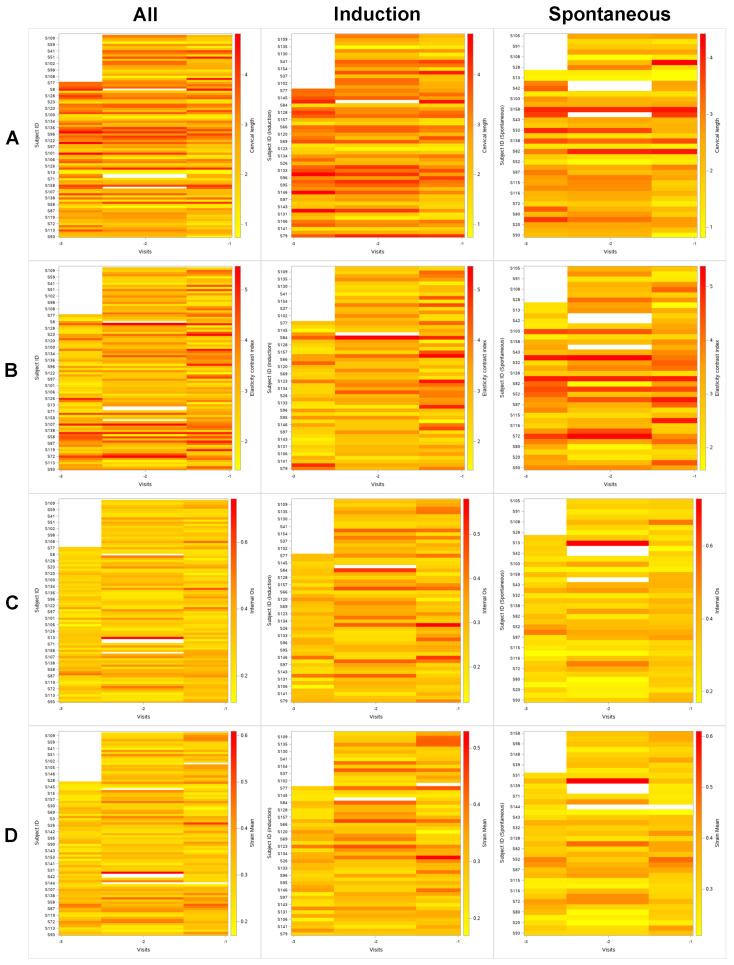
Lasagna plots of the cervical length and the selected E-Cervix parameters against each visit. A lasagna plot is a heatmap for longitudinal data where each subject’s trajectory over time is a horizontal layer, with the simultaneous plotting of trajectories resulting in the stacking of layers, as in lasagna [18]. The horizontal axis of each plot represents a visit counted from the admission for delivery (e.g., −1 being the last visit before admission). The color bar located right to each plot represents each parameter’s value range, red being the highest value, and yellow being the lowest value. The color change from left to right in each picture can be interpreted as change of each parameter’s value at each visit. (**A**): Cervical length; (**B**): Elasticity contrast index; (**C**): Strain mean of the internal os of the cervix; (**D**): Strain mean level; **Left** column, plots including all subjects; **Middle** column, plots of the labor induction group; **Right** column, plots of the spontaneous labor group.

Figure 4 is the lasagna plots of the cervical length and the selected E-Cervix parameters against each visit. The horizontal axis of each plot represents a visit counted from the admission for delivery (e.g., −1 being the last visit before admission). The color bar located right to each plot represents each parameter’s value range, red being the highest and yellow being the lowest. The color change from left to right in each picture can be interpreted as change of each parameter’s value at each visit. For example, middle panel in row A shows the change of the cervical length at each visit in the labor induction group. From the visit −3 to visit −1, the color tends to change from red to yellow which indicates shortening of the cervix toward the admission for delivery.

## 4. Discussion

This longitudinal study showed that the E-Cervix parameters tended to change during the three weeks before the admission for the delivery in women with singleton pregnancies at term. Our results also revealed that there were significant differences in E-Cervix parameters according to the presence or absence of spontaneous labor in these women.

The changes in most E-Cervix parameters, including ECI, IOS, EOS, strain mean, and hardness ratio were in agreement with previous reports. For example, it was previously reported that ECI was significantly higher in patients with a moderately short cervix and who ultimately deliver preterm [16]. In the report, we suggested that ECI values increase with increasing heterogeneity as the uterine cervix ripens. The current data support our suggestion regarding ECI, as ECI increased with advancing gestation irrespective of the development of spontaneous labor. The finding that ECI value was higher in women with spontaneous labor also indicated that there would be more biochemical change and heterogeneity in the cervix of patients with spontaneous labor. Elastographic measurement around the internal os of the cervix has been extensively studied to determine whether it can predict preterm delivery in high- and low-risk pregnancies [20,21,22]. These early reports have all indicated that soft cervix or higher strain values were related to spontaneous preterm delivery. In addition, those values were increased in the spontaneous labor group, as in the case of ECI. All these findings demonstrate that E-Cervix elastography can differentiate the cervical changes according to the time and labor group in term pregnancy. However, there are differences in significance observed among E-Cervix parameters. There may be some difference in sensitivity among E-Cervix parameters in detecting changes of the cervix. For example, in all the E-Cervix parameters except IOS/EOS ratio, there were significant differences between the two labor groups. However, the change according to the days might have been enough to be detected only in several parameters such as ECI, IOS strain, and strain mean. Reproducibility should also be taken into consideration. We previously reported reproducibility in terms of intra- and inter-observer intraclass correlation coefficient (ICC), which ranged between 0.838 and 0.887 for intra-observer ICC and between 0.901 and 0.988 for inter-observer ICC [17]. Although the results showed good to excellent reproducibility, it would be reasonable, and customary to present mean or median values from multiple measurements.

This research is unique in that it is a longitudinal study in which serial measurements were performed so that we can learn the changes of the cervix with regard to the time and the presence or absence of labor around the time of delivery at term. However, there was no abrupt change in any E-Cervix elastographic parameters or cervical length near the onset of labor. The changes seemed to be constant throughout three weeks before admission in both spontaneous labor and labor induction groups (Figure 3). One study has investigated cervical length changes from 17 to 34 weeks and found that the cervical length was predicted to decrease by 0.6 mm per week of advancing gestation, and the changes were linear in singleton pregnancies [23]. According to our data (Table 3), the cervical length is expected to decrease by 1.7 mm per week for three weeks before the admission, and those changes seem to be linear. Although the rate of change was higher than the cervical length change between 17 to 34 weeks, we could not detect accelerated changes in cervical length near the time of admission. Our data provide an insight into the cervical change at term around delivery. The cervix probably continues to change constantly without any further accelerated ripening around the time of birth at least within three weeks from spontaneous labor. We included the labor induction (no labor) group in this study to contrast the cervical changes of the spontaneous labor group with an assumption that there may be “additional” differences in the spontaneous labor group near the time of spontaneous labor. Cervical ripening of the women who are destined to have spontaneous labor is more advanced than that in women who would not.

In a textbook of obstetrics, the process of parturition is divided into four phases, with cervical ripening occurring in phase two of the parturition. The cervix must undergo “extensive remodeling”. The transition from the softening to the ripening phase begins “weeks or days before labor” [24]. However, our data did not support such an argument. The transition from the softening to ripening might have started even earlier.

We enrolled the patients from 36 weeks of gestation because the cervical ripening may ensue before term, and the labor may begin at any time near term. In addition, as the patients should visit every week from 36 weeks of gestation, we thought that it would be best to enroll patients from 36 weeks. Our study implies that cervical ripening near term parturition could be detected by cervical elastographic measurement. Further studies are needed to confirm this. For example, considering that cervical ripening is one of the most important parameters affecting induction failure, cervical elastography could be used to predict successful induction of labor in women with term pregnancies.

There are things to consider when interpreting our results. First, although we assumed a linear relationship between time and variables, it might have been curvilinear if we had performed elastography more frequently. Second, while we presented statistically significant changes in E-Cervix elastography parameters near term pregnancies, the actual differences were relatively small in values to be useful in clinical settings. Finally, as the participating institutions are referral hospitals where patients with high-risk pregnancies are taken care of, this feature is reflected in the subject population. Gestational diabetes mellitus (GDM) comprised 13.6% (13/96), which is quite high, and 5% of patients with hypertensive diseases were included in the labor induction group. When those findings are taken into consideration, the readers should be reminded that the study population in this study was from high-risk patients and showed much heterogeneity.

## Figures and Tables

**Table 1:** Selected E-Cervix parameters.

Measurement Parameter	Description
ECI	ECI score within the ROI, value range: 0 (homogeneity)–81 (heterogeneity)
IOS strain mean level	Standardized strain mean level in 1 cm circle of IOS, value range: 0 (hard)–1 (soft)
EOS strain mean level	Standardized strain mean level in 1 cm circle of EOS, value range: 0 (hard)–1 (soft)
Ratio (IOS/EOS)	IOS strain level/EOS strain level
Strain mean level	Strain mean level within the ROI, value range: 0 (hard)–1 (soft)
Hardness ratio	30-percentile hardness area ratio within the ROI, value range: 0% (soft)–100% (hard)

IOS, internal os of the cervix; EOS, external os of the cervix; ROI, a region of interest; ECI, elasticity contrast index.

**Table 2:** Characteristics and pregnancy outcomes of the participants.

	Spontaneous Labor *n* = 39	Labor Induction *n* = 57	*p*-Value
Maternal age	32.00 (29.00–35.00)	33.00 (31.00–36.00)	0.067
History of abortion	6 (15.38%)	15 (26.32%)	0.220
History of CIN	2 (5.13%)	2 (3.51%)	1.000
History of LEEP	1 (2.56%)	1 (1.75%)	1.000
IVF-ET	7 (17.95%)	13 (22.81%)	0.620
pre-pregnancy BMI	21.77 (20.32–23.50)	21.30 (19.71–23.44)	0.480
Smoking	1 (2.56%)	2 (3.51%)	1.000
Hypertensive diseases	0 (0.00%)	3 (5.26%)	0.270
GDM	6 (15.38%)	7 (12.28%)	0.760
Progesterone use	2 (5.13%)	1 (1.75%)	0.560
Maternal weight at delivery	71.90 (64.70–76.50)	70.30 (65.00–79.90)	0.880
GA at admission	39.57 (39.14–40.29)	39.71 (39.00–40.29)	0.960
GA at delivery	39.57 (39.14–40.43)	39.86 (39.14–40.43)	0.630
Cesarean delivery	3 (7.69%)	21 (36.84%)	0.001
Birthweight	3244 (3000–3404)	3340 (3110–3498)	0.120
NICU admission	2 (5.13%)	1 (1.79%)	0.570

Data are presented as median (interquartile range) or n (%). CIN, cervical intraepithelial neoplasia; LEEP, loop electrosurgical excision procedure; IVF-ET, in vitro fertilization and embryo transfer; BMI, body mass index; GDM, gestational diabetes mellitus; GA, gestational age; NICU, neonatal intensive care unit.

**Table 3:** The results of the regression analyses.

	Days from the Measurement to Admission (α_1_) in Equation (1)	SEM	*p*-Value	Days from the Measurement to Admission (ß_1_) in Equation (2)	SEM	*p*-Value	Labor Group (ß_2_) in Equation (2)	SEM	*p*-Value
Cervical length	−0.02459	0.00483	<0.001	−0.02468	0.00483	<0.001	−0.4810	0.1692	0.0055
ECI	0.0175	0.00824	0.0363	0.01756	0.00824	0.0357	0.2872	0.1386	0.0442
IOS strain mean level	0.00159	0.00072	0.0284	0.001605	0.00072	0.0272	0.0318	0.0122	0.0105
EOS strain mean level	0.001	0.00074	0.1788	0.001033	0.00074	0.1673	0.03131	0.0133	0.0209
IOS/EOS ratio	−0.0004	0.00279	0.8799	−0.00041	0.0028	0.8831	0.02319	0.0418	0.5805
Strain mean	0.00113	0.00053	0.0353	0.001140	0.00053	0.0323	0.03204	0.0103	0.0025
Hardness ratio	−0.2049	0.1142	0.0759	−0.2084	0.1137	0.0699	−6.3078	2.1683	0.0045

SEM, standard error of mean; ECI, elasticity contrast index; IOS, internal os of the cervix; EOS, external os of the cervix; The Equation (1): y= α0 + α1*(days from the measurement to admission); The Equation (2): y = ß0 + ß1*(days from the measurement to admission) + ß2* (labor group), Labor group = 0 in labor induction group, labor group = 1 in spontaneous labor group (i.e., the Equation (2) will be y = ß0 + ß1*(days from the measurement to admission) in labor induction group, and it will be y = (ß0 + ß2)+ ß1*(days from the measurement to admission) in spontaneous labor group). Both α0 and ß0 are constants and not listed.

## Data Availability

The data presented in this study are available on request from the corresponding author. The data are not publicly available due to privacy policy.
